# Multiple piroplasm parasites (Apicomplexa: Piroplasmida) in northeastern populations of the invasive Asian longhorned tick, *Haemaphysalis longicornis* Neumann (Ixodida: Ixodidae), in the United States

**DOI:** 10.1017/S0031182023000914

**Published:** 2023-09

**Authors:** Heidi Herb, Julia González, Francisco C. Ferreira, Dina M. Fonseca

**Affiliations:** 1 Center for Vector Biology, Rutgers University, New Brunswick, NJ 08901, USA; 2 Department of Ecology and Evolution, Rutgers University, New Brunswick, NJ 08901, USA; 3 Department of Entomology, Rutgers University, New Brunswick, NJ 08901, USA

**Keywords:** agriculture, invasive spillover, livestock, One Health, public health, wildlife

## Abstract

Piroplasms, which include the agents of cattle fever and human and dog babesiosis, are a diverse group of blood parasites of significant veterinary and medical importance. The invasive Asian longhorned tick, *Haemaphysalis longicornis*, is a known vector of piroplasms in its native range in East Asia and invasive range in Australasia. In the USA, *H. longicornis* has been associated with *Theileria orientalis* Ikeda outbreaks that caused cattle mortality. To survey invasive populations of *H. longicornis* for a broad range of piroplasms, 667 questing *H. longicornis* collected in 2021 from 3 sites in New Jersey, USA, were tested with generalist piroplasm primers targeting the 18S small subunit rRNA (395–515 bp, depending on species) and the cytochrome b oxidase loci (1009 bp). Sequences matching *Theileria cervi* type F (1 adult, 5 nymphs), an unidentified *Theileria* species (in 1 nymph), an undescribed *Babesia* sensu stricto (‘true’ *Babesia*, 2 adults, 2 nymphs), a *Babesia* sp. Coco (also a ‘true *Babesia*’, 1 adult, 1 nymph), as well as *Babesia microti* S837 (1 adult, 4 nymphs) were recovered. *Babesia microti* S837 is closely related to the human pathogen *B. microti* US-type. Additionally, a 132 bp sequence matching the cytochrome b locus of deer, *Odocoileus virginanus*, was obtained from 2 partially engorged *H. longicornis.* The diverse assemblage of piroplasms now associated with *H. longicornis* in the USA spans 3 clades in the piroplasm phylogeny and raises concerns of transmission amplification of veterinary pathogens as well as spillover of pathogens from wildlife to humans.

## Graphical abstract

**Figure:**
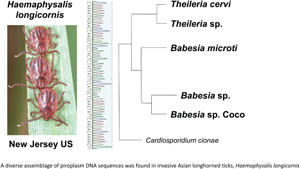

## Introduction

Invasive ticks, mosquitoes and other blood-feeding arthropods may introduce and transmit (i.e. vector) exotic pathogens for which local populations have little or no immunity. Resulting disease can range from mild to severe to fatal and can have a significant impact on human health (e.g. Zika fever, West Nile virus encephalitis), animal health (e.g. redwater fever, blue tongue virus), hasten extinction (e.g. bird malaria) and cause economic damage to agriculture, tourism and other industries (Athni *et al*., 2021). Invasive vectors can also potentially spread existing wildlife pathogens by creating new transmission pathways, which can have significant ecological and public health implications, particularly in the context of One Health (*sensu* Lerner and Berg, 2015).

The phylum Apicomplexa includes well-known blood-borne protozoa such as *Plasmodium falciparum* and *P. vivax*, the primary agents of human malaria (Votýpka *et al*., 2016). The Apicomplexa class Piroplasmida includes *Babesia*, *Theileria* and *Cytauxzoon* that are primarily transmitted by hard ticks (Ixodida: Ixodidae) and can affect a wide range of hosts (Onyiche *et al*., 2021; Almazán *et al*., 2022). While piroplasms were once classified based on morphology and host associations alone, the advent of molecular methods has greatly advanced the overall understanding of the diversity of Piroplasmida (Garrett *et al*., 2019). A recent analysis by Jalovecka *et al*. (2019) indicates that there are at least 10 distinct clades within Piroplasmida, with both *Babesia* spp. and *Theileria* spp. comprising polyphyletic groups in need of taxonomic revision.

*Babesia* spp. are broadly divided into *Babesia* sensu stricto and *Babesia* sensu lato, with the former representing a monophyletic group considered as ‘true *Babesia*’, distinguishable from other piroplasms by their ability to infect the reproductive organs of the tick and to be transmitted to the eggs (transovarial transmission) (Schreeg *et al*., 2016; Jalovecka *et al*., 2019; Schnittger *et al*., 2022). Within *Babesia* sensu lato, one of the best-characterized clades is the *Babesia microti* group, which contains the piroplasms responsible for most human babesiosis cases worldwide, especially in the northern United States (Renard and Mamoun, 2021). Importantly, *B. microti* includes at least 2 different genetic lineages pathogenic to humans and several only known from reservoir hosts such as mice, voles and skunks (Goethert, 2021). Moreover, although still relatively rare, human disease caused by other *Babesia* species such as *B. divergens* in Europe, *B. venatorum* in Asia and Europe and *B. duncani* in North America has been increasingly reported (Scott and Scott, 2018; Hong *et al*., 2019; Kumar *et al*., 2021; Scott *et al*., 2021).

Rates of human babesiosis have been increasing in the USA, particularly in the northeastern states (Almazán *et al*., 2022; Swanson *et al*., 2023). While those infected with *Babesia* may experience fever, chills, headache, muscle aches, fatigue and red or brown urine, some may not have any symptoms at all, especially if their immune systems are not compromised (Almazán *et al*., 2022). People may remain infected for years, and even if asymptomatic, can transmit piroplasms through blood transfusions or organ transplants (Bloch *et al*., 2019), and transmission from infected mothers to developing fetuses has been demonstrated (Horowitz and Freeman, 2020). Piroplasmid infections are typically treated with various combinations of atovaquone, azithromycin, clindamycin and quinine; however, concerns regarding side-effects, drug resistance and drug efficacy indicate the need for development of novel treatment options (Renard and Mamoun, 2021).

Bovine babesiosis caused by *Babesia bigemina* and *Babesia bovis* has long been a concern to American cattle ranchers and several *Theileria* species can sicken horses, cervids and bovids (Almazán *et al*., 2022; Osbrink *et al*., 2022). Both babesiosis and theileriosis cause significant economic losses annually to North American and Australasian agricultural industries due to reduced production, death, abortions, restrictions on animal movement and costs associated with preventive measures and treatments (Dinkel *et al*., 2021; Almazán *et al*., 2022; Osbrink *et al*., 2022; Schnittger *et al*., 2022). Companion animals are also at risk, with infections of *Babesia vulpes*, *Babesia conradae*, *Babesia vogeli*, *Babesia gibsoni* and *Babesia* sp. Coco capable of causing mild to severe disease in dogs in the United States (Dear and Birkenheuer, 2022).

Since the initial discovery of the invasive Asian longhorned tick (*Haemaphysalis longicornis*) in the United States in 2017 (Rainey *et al*., 2018), there have been concerns regarding the potential threats this ectoparasite may pose. In North America, as in Australasia where it expanded to in the early 20th century, *H. longicornis* reproduces asexually (clonally) by parthenogenesis (Schappach *et al*., 2020), which underlies the ability of this species to develop large populations very quickly. In its Australasian range, *H. longicornis* represents a major threat to domestic livestock, heavily parasitizing large ruminants, and impeding production (Heath, 2016).

Globally, *H. longicornis* is a known vector of piroplasms that infect humans, livestock and companion animals, including *B. ovata*, *B. gibsoni*, *B. microti*, *T. uilenbergi* and *T. orientalis* (Li *et al*., 2009; Wu *et al*., 2017; Gray *et al*., 2019; Dinkel *et al*., 2021; Dear and Birkenheuer, 2022) and may also be a vector of *Babesia caballi*, the agent of equine babesiosis (Bautista *et al*., 2001). As in Australia (Marendy *et al*., 2020), in Virginia, USA, *H. longicornis* has already been implicated in the transmission of the virulent *Theileria orientalis* Ikeda genotype that resulted in multiple cattle deaths (Thompson *et al*., 2020; Dinkel *et al*., 2021).

Although humans are not favoured hosts of *H. longicornis*, opportunistic feeding is well documented both in the native and invasive ranges (Bickerton and Toledo, 2020; Wormser *et al*., 2020). In East Asia, *H. longicornis* vectors severe fever with thrombocytopenia syndrome virus (SFTSV), an emerging human tick-borne disease recently reclassified as Dabie bandavirus (Liu *et al*., 2015; Luo *et al*., 2015; Li *et al*., 2021). Under laboratory conditions, US lineages of *H. longicornis* can vector the closely related Heartland virus (Raney *et al*., 2022*a*) as well as Powassan virus (Raney *et al*., 2022*b*), 2 native pathogenic viruses emergent in parts of the USA. They can also vector *Rickettsia rickettsii*, the causative agent of Rocky Mountain spotted fever (Stanley *et al*., 2020). In addition, Bourbon virus has been detected from a larval pool, 2 nymphs and 1 adult field collected *H. longicornis* in Virginia, USA (Cumbie *et al*., 2022). While *H. longicornis* is currently not perceived as a major public health threat in the USA, this status may change given the enormous densities it can reach in favourable habitats (Bickerton and Toledo, 2020; Schappach *et al*., 2020; González *et al*., 2023; Rochlin *et al*., 2023).

The objective of this study was to assess the potential role of *H. longicornis* as a vector of piroplasms in New Jersey (NJ), the most urbanized US state that, maybe surprisingly to many, also boasts the highest density of horses (Rankins and Malinowski, 2020).

## Materials and methods

### Study areas

This study was conducted in 3 sites approximately 1.2 km from each other within the Rutgers University Cook Campus in New Brunswick, NJ (please refer to Ferreira *et al*., 2023 for a map). Surveys for *H. longicornis* were initiated at these sites in 2018 when the species was first detected on a grassy area next to a goat pen (Egizi *et al*., 2019*b*), a site that became known as the ‘Goat Farm’ (40.47444° N, 74.43683° W). The ‘Rutgers Gardens’ site (40.47455° N, 74.42030° W) is inside a 180-acre botanical garden, consisting of designed gardens, plant collections and natural habitats. Finally, the ‘University Inn’ site (40.48413 N, 74.43051 W) is a meadow and forested park behind the Rutgers University Inn & Conference Center. At all sites, local forest is dominated by oak and maple trees and huckleberry and blueberry shrubs (Breden *et al*., 2001), with grassy ecotones.

### Tick surveillance

From June through September 2021, concomitant with surveys for ticks on mammals at the same sites, questing ticks were sampled from 50–75 m^2^ of the vegetation at each site (Ferreira *et al*., 2023). Tick sampling was performed using a white crib flannel sweep measuring 50 × 100 cm with a PVC pipe handle (Egizi *et al*., 2019*b*). The sweep cloth was checked in 1–2 m intervals since *H. longicornis* does not attach firmly to the flannel and often drops off over longer intervals (Bickerton *et al*., 2021). Ticks were collected from both sides of the sweep and identified morphologically in the laboratory to the species level using a stereomicroscope (Leica S8 APO, Leica Microsystems, Deerfield, IL, USA) following appropriate taxonomical keys (Keirans and Litwak, 1989; Egizi *et al*., 2019*a*). The larvae of *H. longicornis* were not stored during these surveys and were not available for pathogen testing. A few questing *H. longicornis* that were found partially engorged (sensu Price *et al*., 2022) were processed separately (see section below on ‘Bloodmeal analysis of partially engorged specimens’).

### DNA extraction and pathogen detection

Each tick was placed in 180 *μ*l of Qiagen buffer ATL with 20 *μ*l Qiagen Proteinase K (10 mg mL^−1^) in microfuge tubes and homogenized with a 5 mm sterile glass bead (Fisher Scientific, Waltham, MA, USA) in a TissueLyser (Qiagen Inc., Valencia, CA, USA). DNA from individual ticks was extracted using Dneasy Blood and Tissue 96-well plate kits (Qiagen Inc., Valencia, CA, USA) following the manufacturer's instructions. DNA was eluted from each column twice with 50 *μ*l of Qiagen's elution buffer AE into separate labelled microtubes.

After reviewing the literature, primers were chosen targeting the multi copy 18S rRNA locus (Table 1) to match a broad range of piroplasm species (Casati *et al*., 2006) and all ticks were tested individually. To further characterize an undescribed new *Babesia* sp. detected, specimens positive for that *Babesia* were also tested using primers targeting the mitochondrial cytochrome oxidase b (*cytb*) locus (Table 1) shown to work across multiple *Babesia* species (Rajapakshage *et al*., 2012).

**Table 1.** Primers used for piroplasm detection in *H. longicornis*

Target	Locus	Primer	Primer sequence (5’ to 3’)	T_a_
*Piroplasmida* spp.	18S rRNA	BJ1	GTCTTGTAATTGGAATGATGG	55°C
		BN2	TAGTTTATGGTTAGGACTACG	
*Babesia* spp.	mtDNA cytochrome b	cytbF1	ATGTTGTCCTATTTGGTTCC	*54–50°C
		cytbR1	ATATGCAAACTTCCCGGCTA	

T_a_ = annealing temperature. The expected 18S rRNA amplicon size varies depending on the species of piroplasm from 395 to 515 bp; the expected cytochrome b amplicon size is 1009 bp.

*Instead of a specific T_a_, a ‘touch-down’ approach was used starting with T_a_ = 54°C and decreasing the T_a_ by 1 degree for 4 additional cycles. This was followed by 40 additional cycles at T_a_ = 52°C.

The targeted loci were amplified in 20 *μ*l reactions with Amplitaq Gold Master Mix (ThermoFisher Scientific, Waltham, MA, USA) following the manufacturer's protocol. After visualizing the amplification in a 1% agarose gel, polymerase chain reactions (PCR) were cleaned with ExoSAP-IT (ThermoFisher Scientific) and Sanger sequenced separately with both primers at Azenta Genewiz (South Plainfield, NJ, USA). The sequences were trimmed and aligned with Geneious Prime 2023.0.1 (Biomatters Inc., San Diego, CA, USA) and the consensus was used as a query in NCBI's Basic Local Alignment Search Tool, BLASTn (Altschup *et al*., 1990).

### Phylogenetic analysis

Consensus sequences were aligned to available NCBI Genbank sequences (Benson *et al*., 2012) representative of the major piroplasm clades (Jalovecka *et al*., 2019) and the alignments were trimmed to the same size (557 bp). Maximum likelihood phylogenetic trees based on the 18S rRNA and *cytb* loci were constructed using IQ-TREE with 1000 ultrafast bootstrap replicates (Nguyen *et al*., 2015; Hoang *et al*., 2018). ModelFinder was used to choose the best-fitting substitution model based on Bayesian information criterion (Kalyaanamoorthy *et al*., 2017). *Cardiosporidium cionae* (GenBank accession number EU052685) and *B. microti* (GenBank accession number NC034637) were used as outgroups for the 18S rRNA and *ctyb* phylogenetic trees, respectively.

### Statistical analysis

To determine whether there are statistically significant differences in infection rates in nymphal *vs* female *H. longicornis*, a binomial generalized linear model using the glm function in R (R Core Team, 2022) was used. To determine the correlation between sample size and infection rates, the lm function in R was used.

### Bloodmeal analysis of partially engorged specimens

DNA from 2 partially engorged *H. longicornis* collected on 15 July 2021 (1 nymph and 1 adult) was isolated using DNeasy Blood and Tissue columns (Qiagen, Valencia, CA, USA). An extraction control was included, and all work was performed in a dedicated clean lab inside a laminar flow hood (Mystaire, Creedmor, NC, USA). Primers CytbVertR1 (5'-GGACGAGGACTATACTACGG-3' from Egizi *et al*., 2013) and BMF1 (5'-AAACTGCAGCCCCTCAGAATGATATTTGTCCTCA-3'), originally called H15149 (Kocher *et al.,* 1989), were used to amplify a 132-nucleotide fragment in the cytochrome oxidase b locus using an annealing temperature of 55°C. A PCR product obtained from the adult *H. longicornis* was purified (ExoSAP-IT, Affymetrix, Santa Clara, CA, USA) then sequenced at Azenta Genewiz (South Plainfield). The sequences were trimmed and aligned with Geneious Prime 2023.0.1 (Biomatters Inc.) and the consensus was used as a query in NCBI's Basic Local Alignment Search Tool, BLASTn (Altschup *et al*., 1990).

## Results

Overall, 667 *H. longicornis* nymphs and adults collected from the environment were screened and evidence of piroplasm parasites was found in 18 ticks (2.7%, Table 2). Adult and nymph infection rates did not differ (infection rates of 2.4 and 2.8%, respectively, *P* value = 0.60). Infection rates among sampling sites reflected sample size (*r*^2^ = 0.92, *P* value <0.01; using adult and nymph data separately to increase the statistical power).

**Table 2.** Numbers of *H. longicornis* nymphs and adults collected from the environment at 3 sites at Rutgers Cook campus and tested for piroplasm DNA (# Pos represents the number of ticks that were positive). Collections were started on 24 June 2021 and proceeded approximately bi-weekly until 10 September 2021 (refer to Ferreira *et al.*, 2023 for details).

	June		July		August		September		Total	
	N	# Pos	N	# Pos	N	# Pos	N	# Pos	N	# Pos
**Goat Farm**	**35**	**2**	**100**	**4**	**14**	**0**	**4**	**0**	**153**	**6**
Nymph	20	2^1^	38	0	7	0	1	0	66	2
Adult	15	0	62	4^1^,^2^,^3^	7	0	3	0	87	4
**Rutgers Gardens**	**26**	**0**	**30**	**0**	**8**	**0**	**0**	**0**	**64**	**0**
Nymph	22	0	16	0	4	0	0	0	42	0
Adult	4	0	14	0	4	0	0	0	22	0
**University Inn**	**78**	**1**	**308**	**10**	**56**	**1**	**8**	**0**	**450**	**12**
Nymph	64	1^3^	250	9^1^,^2^,^3^,^4^,^5^	33	1^1^	7	0	354	11
Adult	14	0	58	1^4^	23	0	1	0	96	1
**Grand Total**	**139**	**3**	**438**	**14**	**78**	**1**	**12**	**0**	**667**	**18**

1 *Babesia microti* S837 (5 positive ticks)

2 *Babesia* sp. Coco (2 positive ticks)

3 *Babesia* sp. (4 positive ticks)

4 *Theileria cervi* (a total of 6 positive ticks)

5 *Theileria* sp. (1 positive ticks)

There was no evidence of piroplasm coinfections (such as double chromatogram peaks) in the positive ticks. The primers targeting the 18S rRNA gene amplified fragments ranging in size from 395 to 515 base pairs (bp) spanning the V4 hypervariable region (Cauvin *et al*., 2019).

**Figure 1.**
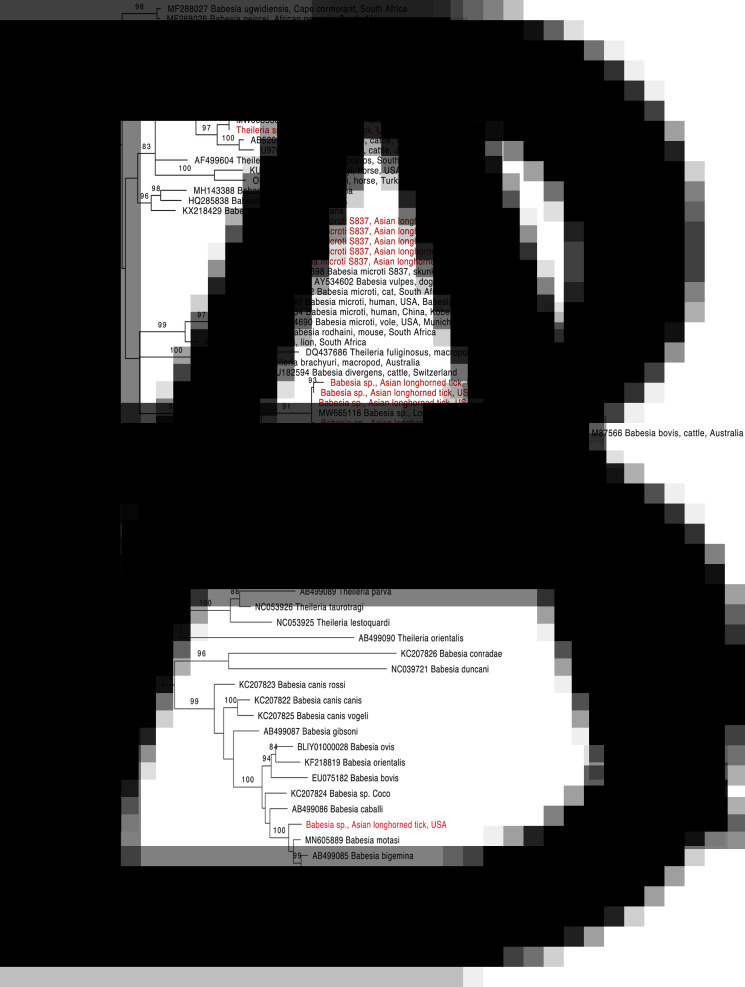
Phylogenetic trees with piroplasm sequences obtained from *Haemaphysalis longicornis* (in red). (a) Tree based on 18S rRNA locus. Constructed with TIM2 + F + I + G4 substitution model. (b) Tree based on cytochrome b locus. Constructed with K3Pu + F + I + G4 substitution model.

The 18S rRNA fragment from a nymph collected at University Inn had a 100% pairwise identity to 5 *Theileria* sp. sequences (GenBank accession numbers MW008536, MW008531, MK262962, MK262963 and MK262959) that are considered ‘Type X’ or ‘divergent’ (Cauvin *et al*., 2019; Olafson *et al*., 2020). This sequence differed by 20 bp (17 mismatches and 3 deletions) from 6 other sequences obtained from *H. longicornis* also collected from the University Inn site. The closest match for these 6 sequences (99.8–100% pairwise identity) was a *Theileria cervi* type F sequence (GenBank U97054). Three of the 6 sequences, 2 from nymphs and 1 from an adult, were identical to U97054 while the remaining 3, all from nymphs, differed by 1 bp (GenBank accession numbers OR612075, OR612078, OR612080). All sequences clustered within a clade containing *T. cervi* and *T. orientalis* (Fig. 1a).

Two 18S rRNA sequences, one from a Goat Farm adult and the other from a University Inn nymph, were identical to a *Babesia* sp. isolate NYT-435 (GenBank accession number MW665118) recently sequenced from a pool of lone star ticks (*Ambylomma americanum*) from Staten Island, NYC (Jain *et al*., 2021). The sequence of the *Babesia* sp. isolate NYT-435 was also the closest match for 2 other sequences, 1 from another Goat Farm adult and a second University Inn nymph (99.7 and 99.5% pairwise identities with a 1 and 2 bp difference, respectively; GenBank accession numbers OR612068, OR612072, respectively). The next best match for these 4 sequences (97.9–98.2% pairwise identity) was a *Babesia* sp. from a white-tailed deer in Texas (GenBank accession number HQ264120). These 4 sequences clustered within the *Babesia* sensu stricto clade with *B. bovis* and *Babesia ovis* (Fig. 1a). From one of the specimens that were positive for the unknown *Babesia*, a 1009 bp DNA fragment was amplified with the *cytb* primers (GenBank accession number OR610156). The closest match to this sequence was *Babesia motasi* isolate Lintan (GenBank acc. num. MN605889) with 90.8% pairwise identity. The sequence from this *H. longicornis* clustered with *B. motasi* in the *Babesia* sensu stricto clade (Fig. 1b).

The 18S rRNA from 2 sequences, one from a University Inn nymph and another from a Goat Farm adult, matched *Babesia* sp. Coco. The sequence from the Goat Farm adult was 100% pairwise identical to Genbank EU109716, whereas the sequence from the University Inn nymph differed by 1 bp and had a 99.8% pairwise identity (GenBank accession number OR612082).

Finally, 5 18S rRNA sequences, from 2 nymphs and 1 adult from the Goat Farm and 2 nymphs from the University Inn, had a 100% pairwise identity to *B. microti* isolate S837 (GenBank accession number AY144698). In the phylogenetic tree, these sequences clustered with *B. vulpes* within the *B. microti*-like group (Fig. 1a; Jalovecka *et al*., 2019).

A 132 bp DNA fragment was amplified and sequenced from a partially engorged adult *H. longicornis* and was 100% identical to a cytb fragment from white-tailed deer, *Odocoileus virginianus* (GenBank accession number AF535863).

## Discussion

DNA sequences belonging to 3 piroplasm clades were detected in 2.7% of field-collected *H. longicornis* nymphs and adults in NJ, United States. Infection rates for *Theileria*, *Babesia* sensu stricto and *B. microti*, were 1, 0.9 and 0.8%, respectively. While these may be low piroplasm infection rates compared to studies that detected *T. orientalis* in 12.7% of *H. longicornis* in Virginia (Thompson *et al*., 2020, 2022), those were collected from the cattle farm where the first US outbreak of *T. orientalis* Ikeda occurred, which would have increased the likelihood that local vectors were infected. Overall, there have been few exploratory examinations of piroplasms in *H. longicornis* in the United States.

This is the first report of *Theileria* species besides *T. orientalis* Ikeda in *H. longicornis* in the United States. Most sequences matched *T. cervi* type F, a piroplasm that commonly infects deer and other cervids (Cauvin *et al*., 2019; Olafson *et al*., 2020). However, 1 sequence differed in at least 20 bp from *T. cervi* type F and instead matched a strain denoted ‘divergent’ or ‘type X’ found in wild and farmed deer in Florida and in *Anocenter nitens*, the tropical horse tick, parasitizing white-tailed deer in Texas (Cauvin *et al*., 2019; Olafson *et al*., 2020). US populations of *H. longicornis* have often been reported feeding on white-tailed deer (Tufts *et al*., 2021), and finding deer DNA in a partially engorged tick supports this. These findings indicate that *H. longicornis* may be involved in the transmission cycle of *Theileria* in NJ.

This is also the first report of *Babesia* in questing un-engorged *H. longicornis* in the United States. Specifically, the phylogenetic analyses using both 18S and cytochrome b loci indicate the unknown *Babesia* sequences fall within the *Babesia* sensu stricto clade. *Babesia* sensu stricto (also referred to as ‘true *Babesia*’) are pathogens of both veterinary and medical importance capable of transovarial transmission in their tick vectors, allowing the parasite to propagate in the absence of vertebrate reservoirs (Jalovecka *et al*., 2019).

*Babesia* sp. Coco, another ‘true *Babesia*’ was also detected in 2 *H. longicornis. Babesia* sp. Coco can be pathogenic to dogs but usually only if they are immunocompromised (Birkenheuer *et al*., 2004; Holman *et al*., 2009; Sikorski *et al*., 2010; Dear and Birkenheuer, 2022), so it is unclear whether dogs are incidental hosts or are an important reservoir species that manifest clinical disease when immunocompromised. Dogs are considered an important blood host for *H. longicornis* in the United States (Trout Fryxell *et al*., 2021; Thompson *et al*., 2022).

Finally, this is the first report of *B. microti* genotype S837 in *H. longicornis.* This genotype is found in skunks *Mephitis mephitis* (Goethert, 2021), which are an important host for this tick species in NJ (Ferreira *et al*., 2023) and, critically, *H. longicornis* has been shown to be a competent vector of *B. microti* under experimental conditions (Wu *et al*., 2017). Of note, there is an overall lack of knowledge of the biology and epidemiology of wildlife piroplasms in the northeastern USA. In areas endemic for human babesiosis, molecular studies of wildlife piroplasms rarely employ sequencing to confirm parasite identity, although piroplasm parasites thought to only infect wildlife and/or domestic animals have recently been reported infecting humans in North America (Scott *et al*., 2021) and elsewhere (Hong *et al*., 2019).

Although the health risk of *H. longicornis* to US livestock was established by the outbreak of *T. orientalis* Ikeda in cattle in the state of Virginia (Thompson *et al*., 2020; Dinkel *et al*., 2021), there is limited research regarding the veterinary and medical significance of *H. longicornis* in the USA, which has focused primarily on testing the ability of US specimens to transmit pathogens of known public health concern (Breuner *et al*., 2020; Stanley *et al*., 2020; Raney *et al*., 2022*a*). The discovery of various piroplasm parasites in questing *H. longicornis* specimens in NJ brings forth new concerns. As a result, it becomes crucial to prioritize studies that delve into the role of *H. longicornis* as an actual vector for these pathogens.

## Data Availability

Nucleotide sequence data reported in this paper are available in GenBank™, EMBL and DDBJ databases under the accession numbers OR610156 and OR612065-OR612082.
